# Rapid Detection of SARS-CoV-2 RNA Using Reverse Transcription Recombinase Polymerase Amplification (RT-RPA) with Lateral Flow for N-Protein Gene and Variant-Specific Deletion–Insertion Mutation in S-Protein Gene

**DOI:** 10.3390/v15061254

**Published:** 2023-05-26

**Authors:** Jose L. Malaga, Monica J. Pajuelo, Michiko Okamoto, Emmanuel Kagning Tsinda, Kanako Otani, Pablo Tsukayama, Lucero Mascaro, Diego Cuicapuza, Masamichi Katsumi, Kazuhisa Kawamura, Hidekazu Nishimura, Akie Sakagami, Yo Ueki, Suguru Omiya, Satoshi Okamoto, Asami Nakayama, Shin-ichi Fujimaki, Chuyao Yu, Sikandar Azam, Eiichi Kodama, Clyde Dapat, Hitoshi Oshitani, Mayuko Saito

**Affiliations:** 1 Department of Virology, Tohoku University Graduate School of Medicine, Sendai 980-8575, Japan; joseluis.malagagranda@gmail.com (J.L.M.);; 2 Laboratorio Microbiología Molecular, Laboratorios de Investigación y Desarrollo, Facultad de Ciencias y Filosofía, Universidad Peruana Cayetano Heredia, Lima 15102, Peru; 3 Center for Biomedical Innovation, Sinskey Lab, Massachusetts Institute of Technology, Cambridge, MA 02139, USA; 4 National Institute of Infectious Diseases, Tokyo 162-8640, Japan; 5 Laboratorio de Genómica Microbiana, Universidad Peruana Cayetano Heredia, Lima 15102, Peru; 6 Sendai City Institute of Health, Sendai 984-0002, Japan; 7 Sendai Shirayuri Women’s College, Sendai 981-3107, Japan; 8 Kawamura Children’s Clinic, Sendai 981-0907, Japan; 9 Virus Research Center, Clinical Research Division, Sendai Medical Center, Sendai 983-8520, Japan; 10 Department of Microbiology, Miyagi Prefectural Institute of Public Health and Environment, Sendai 983-0836, Japan; 11 Department of Clinical Laboratory, Tohoku Kosai Hospital, Sendai 980-0803, Japan; 12 Department of Laboratory Medicine, Tohoku University Hospital, Sendai 980-8574, Japan; 13 International Research Institute of Disaster Science, Tohoku University, Sendai 980-8572, Japan; 14 WHO Collaborating Centre for Reference and Research on Influenza, The Peter Doherty Institute for Infection and Immunity, Melbourne, VIC 3000, Australia

**Keywords:** SARS-CoV-2, variant of concern (VOC), deletion–insertion mutation, COVID-19, recombinase polymerase amplification (RPA)

## Abstract

Rapid molecular testing for severe acute respiratory coronavirus 2 (SARS-CoV-2) variants may contribute to the development of public health measures, particularly in resource-limited areas. Reverse transcription recombinase polymerase amplification using a lateral flow assay (RT-RPA-LF) allows rapid RNA detection without thermal cyclers. In this study, we developed two assays to detect SARS-CoV-2 nucleocapsid (N) gene and Omicron BA.1 spike (S) gene-specific deletion–insertion mutations (del211/ins214). Both tests had a detection limit of 10 copies/µL in vitro and the detection time was approximately 35 min from incubation to detection. The sensitivities of SARS-CoV-2 (N) RT-RPA-LF by viral load categories were 100% for clinical samples with high (>9015.7 copies/µL, cycle quantification (Cq): < 25) and moderate (385.5–9015.7 copies/µL, Cq: 25–29.9) viral load, 83.3% for low (16.5–385.5 copies/µL, Cq: 30–34.9), and 14.3% for very low (<16.5 copies/µL, Cq: 35–40). The sensitivities of the Omicron BA.1 (S) RT-RPA-LF were 94.9%, 78%, 23.8%, and 0%, respectively, and the specificity against non-BA.1 SARS-CoV-2-positive samples was 96%. The assays seemed more sensitive than rapid antigen detection in moderate viral load samples. Although implementation in resource-limited settings requires additional improvements, deletion–insertion mutations were successfully detected by the RT-RPA-LF technique.

## 1. Introduction

The coronavirus disease 2019 (COVID-19) pandemic caused by severe acute respiratory coronavirus 2 (SARS-CoV-2) has led to a global health crisis in the last century. By May 2023, over 765.2 million cases and 6.9 million deaths were reported, and challenges to public health still prevail. The Omicron (B.1.1.529) variant was first reported in Africa in November 2021 [1] and rapidly became a worldwide dominant variant of concern (VOC) by early 2022 [2]. Consequently, the Omicron subvariants emerged and the global prevalence of the subvariants is reported as half for XBB.1.5 (47.5%), followed by XBB.1.16 (8.6%), XBB.1.9.1 (12.4%), XBB.1.9.2 (3.8%), XBB.2.3 as of 7 May 2023 [3]. XBB.1.5 is the most reported subvariant in American and European regions, while XBB.1.16 and XBB.1.9.1 are dominating Southeast Asia and Eastern Mediterranean regions, respectively. In the African and Western Pacific regions, similar proportions of different XBB subvariants are circulating. However, a reduction in testing and genomic surveillance challenges the assessment of the subvariants, which may be of particular concern in low-resource settings. 

Omicron (B.1.1.529) has > 30 mutations on the spike gene (S) [4]. It is characterized by the presence of three deletions (del69, del143-145, del211) and insertion 214 (ins214) in the N-terminal domain. Deletion del143-145 promotes immune evasion [5] and ins214 in combination with Y145D shows a seven-fold resistance to antibodies [6]. Mutations D614G+P681H are known to promote TMPRSS2-independent viral entry, which probably increased the incidence of COVID-19 cases with upper respiratory tract infection [6]. The Omicron subvariant XBB.1.5, a variant of interest (VOI), has a specific mutation of S486P associated with increased ACE2 affinity [7]. Recently, XBB1.16 (VOI) with additional mutations E180V, F486P, and K478R has been spreading without additional public health risk to XBB1.5 [8]. 

Developing sensitive and specific diagnostic tests is crucial for identifying infected persons to isolate and intervene in the transmission spread [9,10]. Molecular diagnosis using real-time reverse transcription polymerase chain reaction (real-time RT-PCR) has been widely used for COVID-19 diagnosis and is mainly based on the detection of the partial nucleocapsid (N) protein region. Whole viral genome sequencing has also been conducted to monitor variants of concern (VOCs), such as Alpha, Beta, Delta, and Omicron, and to understand viral evolution patterns [11]. Despite the implementation of the COVID-19 vaccination program, the emergence of new variants and subvariants has led to several surges in cases [12].

Although next-generation sequencing (NGS) is widely used at an unprecedented level, the coverage of genomic surveillance based on sequencing remains variable among countries, especially in low- and middle-income countries owing to limited resources [13]. Mutation-specific real-time RT-PCR assays have also been implemented for the detection of VOCs [14,15,16], thus reducing the time and cost of VOC monitoring. SARS-CoV-2 antigen rapid detection test (Ag-RDT) possesses favorable characteristics of low-cost, point-of-care, and rapid testing. However, it has relatively low sensitivity in clinical samples with low or moderate viral loads [17,18]. To the best of our knowledge, this method has not yet been adapted to detect specific VOCs. 

Several isothermal nucleic acid amplification detection systems have been recognized for their utility, especially in point-of-care diagnosis [19]. Reverse transcription loop-mediated isothermal amplification (RT-LAMP) and reverse transcription recombinase polymerase amplification (RT-RPA) are sensitive, specific, and rapid alternatives for the detection of SARS-CoV-2 [20]. In particular, RPA has key features, such as a simple primer design, no initial heating step, low and constant operational temperature, multiplex capability, and enhanced tolerance to inhibitors [21,22]. Two rapid molecular studies using a multi-step procedure for the detection of Delta-specific mutation R203M and three other VOC-specific mutations showed high accuracy in clinical samples taking about 60 min [23,24]. Similarly, a single-copy sensitive assay for the Delta mutation L452R using a two-step procedure showed a reaction time of 75 min [25]. Therefore, a single-step assay with less reaction time would be useful.

In this study, we developed two single-step rapid RT-RPA-LF assays for the detection of the SARS-CoV-2 N gene and the Omicron BA.1-specific deletion 211 and insertion 214 (del211/ins214) of the spike (S) gene in less than 35 min. Its diagnostic characteristics were evaluated in vitro and in vivo using 454 clinical samples classified according to different viral loads and a wide range of SARS-CoV-2 variants obtained from Japan and Peru. 

## 2. Materials and Methods

### 2.1. Primer and Probe Design

#### 2.1.1. Nucleocapsid Phosphoprotein Gene

For the diagnosis of SARS-CoV-2, specific RPA primers were designed to amplify the 166 nucleotide (nt) segment of the SARS-CoV-2 N gene (GenBank: LC523807.1). Primer BLAST [26] analysis was performed using the TwistAmp DNA Amplification Kit Assay Design Manual [27]. A 46 nt long probe was designed to have a fluorescein isothiocyanate molecule (6-FITC) at the 5′ end, an abasic site (d-pacer) at probe nucleotide position 31, and a C3-dSpacer at the 3′ end (Table S1). The reverse primer was modified to include a biotin molecule at the 5′ end. The primers and probes were synthesized by FASMAC (Kanagawa, Japan).

#### 2.1.2. Omicron BA.1 (del211/ins214), Spike Glycoprotein Gene

**Figure 1:**
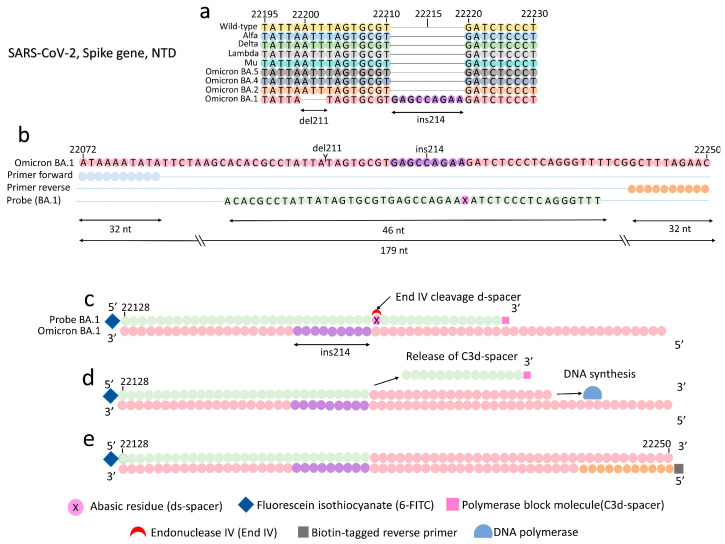
Principle for Omicron BA.1 (S) RT-RPA-LF. (**a**) Representation of the del211/ins214 located in the N-terminal domain (NTD), spike gene, Omicron BA.1. (**b**) RPA primers amplify a 179 bp segment, and a 46 nt long probe anneals a segment along the del211/ins214. (**c**) Endonuclease IV specifically cleaves the d-spacer after the alignment of the probe and del211/ins214. (**d**) The C3-spacer is released from the probe. DNA polymerase incorporates nucleotides from the 5′ to the 3′ end. (**e**) A 179 bp amplicon is synthesized containing a 6-FITC tag at the 5′ end and a biotin at the 3′ end. The doubled tag RPA amplicon can be detected by the universal lateral flow strips specific for 6-FITC/biotin molecules.

We selected the Omicron BA.1-specific deletion–insertion mutation, del211/ins214, in the S gene for primer and probe design (Figure 1a). The prevalence of del211/ins214 in 687 whole genome sequences of BA.1 reported in GISAD [28] from 15 January to 17 February 2022 was 100%; the Pangolin COVID-19 Lineage Assigner [29] was used. Multiple sequence alignment was performed among 57 BA.1 sequences and the SARS-CoV-2 reference strain (MN908947.3) using MEGA Version 7.0 [30]. A 394-nucleotide consensus sequence of Omicron BA.1 was named “S_211del+214ins_deleted” (Table S2) and used to design SARS-CoV-2-specific primers. In addition, the probes were designed by aligning a 46 nt segment covering del211/ins214 (Figure 1b). A probe included a fluorescein isothiocyanate molecule (6-FITC) at the 5′ end, an abasic residue (d-spacer) located after the ins214, and a C3-dSpacer at the 3′ end. It was expected that annealing of the probe to the del211/ins214 segment would allow the endonuclease IV to cleave the abasic residue (d-spacer) (Figure 1c), releasing the C3-dSpacer (Figure 1d) and allowing the DNA polymerase to synthesize complementary DNA from the 5′ to the 3′ end (Figure 1e). The reverse primer included a biotin molecule at the 5′ end. Primers and probes were synthesized by FASMAC. The primer and probe are evaluated in Appendix A. The selected primers and probes are listed in Table S1.

### 2.2. Isothermal Amplification Using RT-RPA

#### 2.2.1. SARS-CoV-2 Partial Nucleocapsid Gene

We developed a one-step isothermal SARS-CoV-2 RNA amplification and detection method using the TwistAmp Basic RPA Kit (TwistDX, Cambridge, UK), reverse transcriptase Moloney Murine Leukemia Virus enzyme (M-MLV) (Invitrogen, Carlsbad, CA, USA), and endonuclease IV (New England Biolabs, Ipswich, MA, USA). The mixture comprised rehydration buffer (29.5 µL), nuclease-free water (6 µL), forward primer (RPA_N_2F) (360 nM) (Table S1), reverse biotin-labeled primer (RPA_N_2R-Bio) (360 nM), M-MLV (4 U/µL), dithiothreitol (DTT) (2 mM) (Invitrogen), endonuclease IV (0.1 U/µL), RNAaseOUT^TM^ (0.4 U/µL) (Invitrogen), and RPA_Probe_N1 (40 nM). The mixture was transferred to a microtube containing pellet enzymes provided with the RPA Kit. Magnesium acetate (14 nM) was then carefully applied to the inner part of the tube lid. Finally, 5 µL of an RNA template was added to the mixture, resulting in a final volume of 50 µL. The reaction tube was centrifuged for 5 s, vortexed for 3 s, and centrifuged again. The mixture was incubated at 37 °C for 30 min using a block heater CTU-N (Taitec Co., Ltd., Tokyo, Japan). We performed gentle hand-mixing and centrifuged the mixture after 5 min of incubation. 

#### 2.2.2. Omicron BA.1 (del211/ins214)

For the amplification of the Omicron BA.1, the master mix consisted of rehydration buffer (29.5 µL), nuclease-free water (4.6 µL), forward primer (RPA_214ins_SET2_F) (480 nM) (Table S1), reverse biotin-labeled primer (RPA_214ins_SET2_R_Bio) (480 nM), M-MLV (4 U/µL), DTT (2 mM), endonuclease IV (0.1 U/µL), RNAaseOUT^TM^ (0.4 U/µL), and RPA_214INS_Probe_2 (60 nM), and the mixture was transferred to a microtube containing the pellet enzymes provided in the RPA Kit. Magnesium acetate (14 nM) was then carefully applied to the inner part of the tube lid. Finally, 5 µL of an RNA template was added to the mixture, resulting in a final volume of 50 µL. The reaction tube was centrifuged for 5 s, vortexed for 3 s, and centrifuged again. The mixture was incubated at 37 °C for 30 min in a block heater. Soft-hand mixing and spin-down were performed after the first five minutes of incubation.

### 2.3. Detection of Amplicons Using Lateral Flow Strips

The 6-FITC- and biotin-labeled amplicons generated by both SARS-CoV-2 (N) RT-RPA-LF and Omicron BA.1 (S) RT-RPA-LF were detected using HybriDetect lateral flow strips (Milenia Biotec, Gieben, Germany). In a tube, 2 µL of RPA amplicon was carefully mixed with 98 µL of assay buffer. The lateral flow strip was then immersed in the buffer, and positive results were considered if a band was confirmed within 5 min, as previously described [31].

### 2.4. Limit of Detection and Cross-Reactivity

To assess the detection limit, we tested 10-fold dilutions of the RNA standards with concentrations equivalent to 10^4^, 10^3^, 10^2^, and 1 copies/µL. The standards consisted of a 166-nucleotide segment of the N gene flanked by RPA primers for SARS-CoV-2 (N) RT-RPA-LF (Appendix B). For Omicron BA.1 (del211/ins214) detection, RNA standards corresponded to a 185 nt segment of the S gene containing the del211/ins214 (Appendix B). A synthetic segment of 395 bp of the wild-type S gene flanked by the Omicron BA.1 primer was used as a negative control (Appendix C). Nuclease-free water was used as the non-template control (NTC). The resulting amplicons were analyzed using lateral flow strips and 3% gel electrophoresis. To evaluate the cross-reactivity of the SARS-CoV-2 (N) RT-RPA-LF and Omicron BA.1 (S) RT-RPA-LF assays, we tested the standards (ATCC, Manassas, VA, USA), virus isolates, and clinical samples associated with some common human respiratory viruses (Table S3).

### 2.5. Clinical Samples

**Figure 2:**
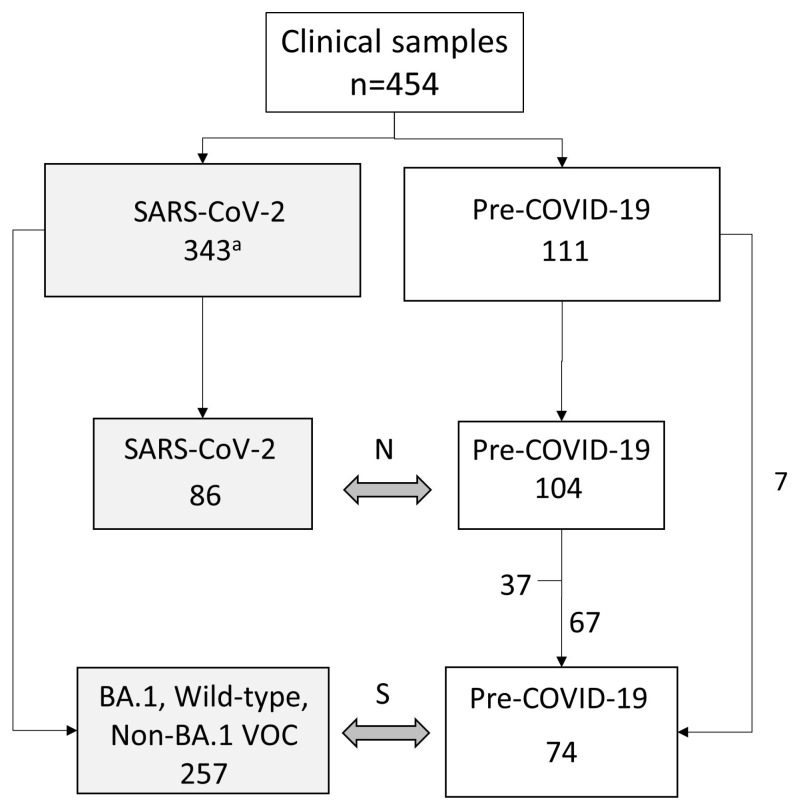
Study design diagram. N: SARS-CoV-2 (N) RT-RPA-LF, S: Omicron BA.1 (S) RPA-LF, VOC: Variant of concern. a. The sample numbers by institutions are described in Table S4.

**Figure 3:**
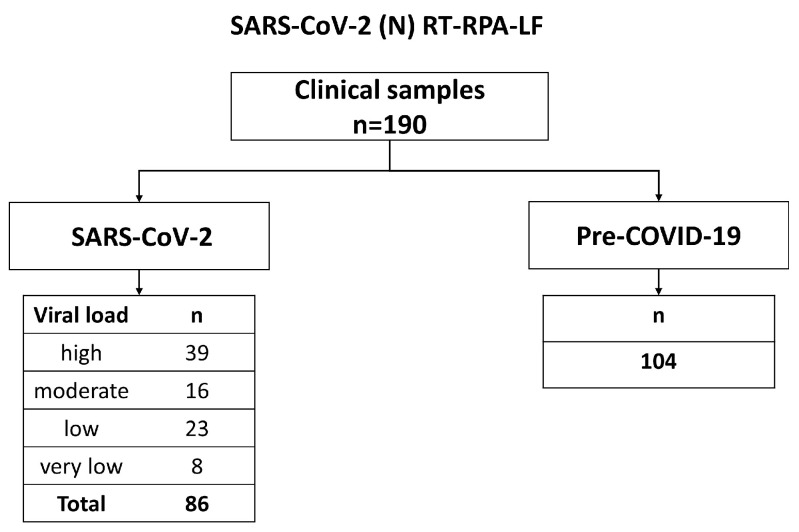
Flow chart for the diagnostic evaluation of SARS-CoV-2 (N) RT-RPA-LF.

**Figure 4:**
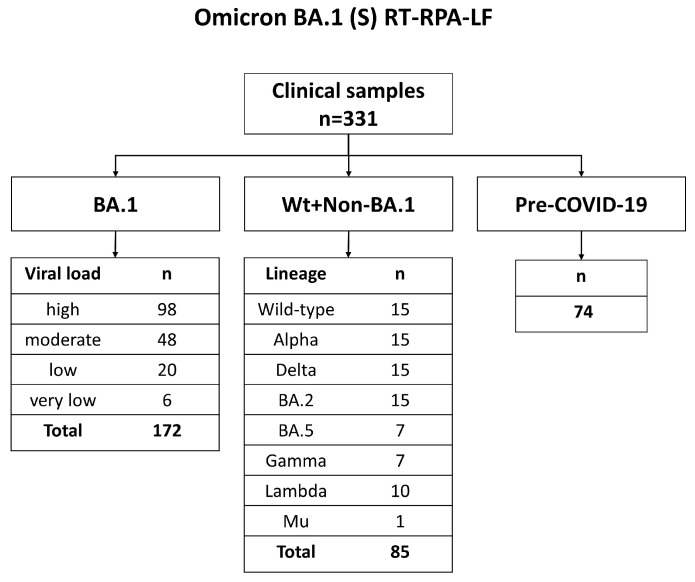
Flow chart for the diagnostic evaluation of Omicron BA.1 (S) RT-RPA-LF. Wt: Wild type. Viral loads were classified as a high viral load: >9015.7 copies/µL, moderate viral load: 385.6–9015.7 copies/µL, low viral load: 16.5–385.5 copies/µL, and very low viral load: <16.5 copies/µL.

We obtained 454 clinical respiratory samples from two hospitals and three laboratories in Miyagi, Japan, and Peru (Figure 2, Table S4). One hundred eleven nasopharyngeal samples were collected from children with respiratory symptoms before the COVID-19 pandemic. Among 328 SARS-CoV-2-positive samples, 86 were included for the evaluation of SARS-CoV-2 (N) RT-RPA-LF (Figure 3), and 257, including variants of BA.1 (n = 172), BA.2 (n = 15), BA.5 (n = 7), Alpha (n = 15), Delta (n = 15), Gamma (n = 7), Lambda (n = 10), and Mu (n = 1), were tested to evaluate Omicron BA.1 (S) RT-RPA-LF (Figure 4). Sixty-seven pre-pandemic samples were tested in both RT-RPA-LF assays. 

SARS-CoV-2 variants were identified based on Sanger sequencing (partial S gene, Table S6) or whole genome sequencing. The SARS-CoV-2 sequences collected in Japan (n = 224) and included in the Omicron BA.1 (S) RT-RPA-LF testing have been submitted to GISAID [28], and they can be identified using the following accession numbers: EPI_ISL_13208893, EPI_ISL_13209110, EPI_ISL_14110653, EPI_ISL_14125573 to EPI_ISL_14125575, EPI_ISL_14139800, EPI_ISL_14139805, EPI_ISL_14139806, EPI_ISL_17263993 to EPI_ISL_17264007, EPI_ISL_17264009 to EPI_ISL_17264089, EPI_ISL_17264091 to EPI_ISL_17264208, and EPI_ISL_17267731 to EPI_ISL_17267732. The genetic sequences from samples collected and previously submitted in Perú (n = 33) were accessed from GISAD to assess the presence of del211/ins214 and establish the group wildtype and non-BA.1 VOC. The sequences can be identified following the accession numbers: EPI_ISL_5146989, EPI_ISL_5146994, EPI_ISL_5147024, EPI_ISL_5147025, EPI_ISL_5147044, EPI_ISL_5147060, EPI_ISL_5147099 to EPI_ISL_5-147101, EPI_ISL_5147103, EPI_ISL_5147104, EPI_ISL_5147146, EPI_ISL_5147152, EPI_I- SL_5147665, EPI_ISL_7961247, EPI_ISL_7961268, EPI_ISL_7961291, EPI_ISL_7961304, E-PI_ISL_7961310, EPI_ISL_7961333, EPI_ISL_7961339, EPI_ISL_7961347, EPI_ISL_7961373, EPI_ISL_7961375, EPI_ISL_7961381, EPI_ISL_7961473 to EPI_ISL_7961476, EPI_ISL_96-37468, EPI_ISL_9637469, EPI_ISL_9637471 and EPI_ISL_9637473. 

### 2.6. RNA Extraction

RNA was extracted using the QIAmp Viral RNA Mini Kit (Qiagen, Valencia, CA, USA), MagMAX CORE Nucleic Acid Purification Kit (ThermoFisher Scientific, Paisley, UK), Maxwell RSC Total Nucleic Acid Purification Kit (Promega, Madison, WI, USA), and Nucleic Acid Extraction Purification Kit (Sansure Biotech, Changsha, China). The samples were tested for SARS-CoV-2 using real-time RT-PCR [32,33] and stored at −80 °C until further use.

### 2.7. Diagnostic Evaluation of RT-RPA-LF in Clinical Samples 

#### 2.7.1. SARS-CoV-2 (N) RT-RPA-LF 

A total of 190 clinical samples were tested using the SARS-CoV-2 (N) RT-RPA-LF assay. The samples were divided into a “SARS-CoV-2” group (n = 86), consisting of samples that tested positive using SARS-CoV-2 RT-qPCR, and a “Pre-COVID-19” group (n = 104), consisting of samples collected before the COVID-19 pandemic. Two technicians who evaluated the RT-RPA-LF were blinded to the SARS-CoV-2 real-time RT-PCR results. An in vitro transcribed RNA standard (10^5^ copies/µL) (Appendix C) was used as a positive control, and nuclease-free water was used as the NTC in all experiments. 

Viral loads were classified in the following way. High viral load: >9015.7 copies/µL, moderate viral load: 385.6–9015.7 copies/µL, low viral load: 16.5–385.5 copies/µL, and very low viral load: <16.5 copies/µL.

#### 2.7.2. Omicron BA.1 (S) RT-RPA-LF

We tested 331 clinical samples using the Omicron BA.1 (S) RT-RPA-LF assay. Based on the results of Sanger sequencing or NGS, we created a “BA.1” group (n = 172) consisting of samples confirmed to be Omicron BA.1. The samples were selected according to the cycle quantification (Cq) level categories described above (Figure 4). We established two negative groups: The “Wt+Non-BA.1” group (n = 85), consisting of samples confirmed as wild type and VOCs other than BA.1, and the “Pre-COVID-19” group, consisting of respiratory samples collected before the COVID-19 pandemic (n = 74) (Figure 3). The test was blinded, as explained above. Transcribed RNA standard containing the del211/ins214 (10^5^ copies/µL) was used as a positive control (Appendix C), and a plasmid containing the wild type for del211/ins214 (10^5^ copies/µL) was used as a negative control. Nuclease-free water was used as the NTC. 

#### 2.7.3. Test Accuracy Calculations

Sensitivity was classified according to different viral load categories in the following way. High viral load: >9015.7 copies/uL, moderate viral load: 385.5–9015.7 copies/uL, low viral load: 16.5–385.5 copies/uL, and very low viral load: ˂16.5 copies/uL (Figure 2 and Figure 3). These categories corresponded to Cq ˂ 25, Cq: 25–29.9, Cq: 30–34.9, and Cq: 35–40, respectively, determined using the correlation curve of the viral load (copies/uL) against Cq of real-time RT-PCR. The 95% confidence interval (95% CI) for sensitivity and specificity was calculated using the Wilson–Brown method in GraphPad Prism version 9.4.1 for Windows (GraphPad Software, San Diego, CA, USA).

### 2.8. Mutation Frequency of del211/ins214 in the Tested Clinical Samples

The clinical samples tested in Omicron BA.1 (S) RT-RPA-LF were classified based on the Pangoline classification using the Pangolin COVID-19 Lineage Assigner as described above. We created a subset of the SARS-CoV-2 sequence data from the clinical samples with BA.1 (n = 172) and “wild-type+non-BA.1 VOC” (n = 85) (Figure 4), and multiple alignments were performed against the reference strain NC.045512.2 using MEGA, Version 7.0 [30]. The presence of del211 and ins214 was visually evaluated, and their prevalence percentage was reported. 

## 3. Results

**Figure 5:**
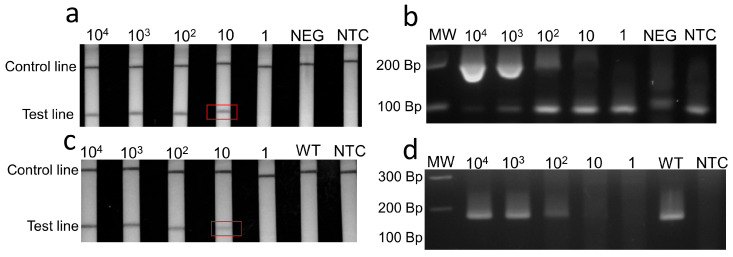
The detection limit of SARS-CoV-2 (N) RT-RPA-LF and Omicron BA.1 (S) RT-RPA-LF based on RNA standards. (**a**,**b**), SARS-CoV-2 (N) RT-RPA-LF showed a detection limit up to 10 copies/µL of RNA standards. (**c**,**d**) The Omicron BA.1 (S) RT-RPA-LF showed a detection limit of 10 copies/µL of RNA standards. The detection limits are marked in the red frames. WT: Wild-type S gene (10^4^ Copies/µL). NEG: negative control, human DNA. NTC: Non-template control, nuclease-free water. MW: Molecular weight marker. Bp: Base pairs.

**Figure 6:**
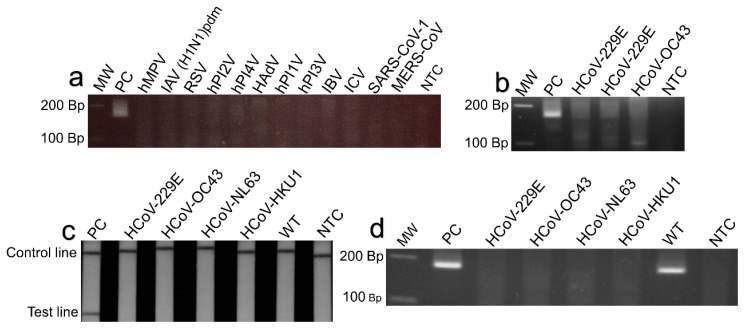

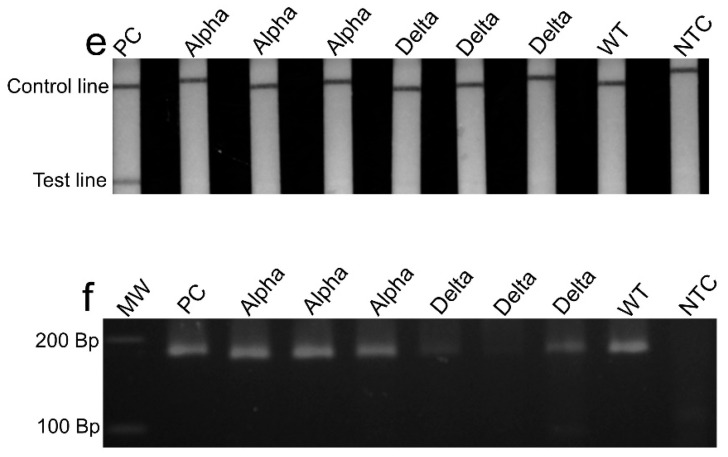
Cross-reactivity of SARS-CoV-2 (N) RT-RPA-LF tested against nucleic acid samples from common respiratory pathogens; (**a**) no cross-reactivity was observed for the SARS-CoV-2 (N) RT-RPA-LF testing human metapneumovirus (hMPV), influenza A virus H1N1 pandemic (IAV H1N1-pdm), respiratory syncytial virus (RSV), human parainfluenza virus type 2 (hPI2V), human parainfluenza virus type 4 (hPI4V), human adenovirus (HAdV), human parainfluenza virus type 1 (hPI1V), human parainfluenza virus type 3 (hPI3V), influenza B virus (IBV), influenza C virus (ICV), severe acute respiratory syndrome coronavirus (SARS-CoV-1), and Middle East respiratory syndrome coronavirus (MERS-CoV); (**b**) no cross-reactivity was observed testing human coronavirus 229E (HCoV-229E) and human coronavirus OC43 (HCoV-OC43). (**c**,**d**) No cross-reactivity was observed for the Omicron BA.1 (S) RT-RPA-LF testing pre-COVID-19 coronaviruses 229E (HCoV-229E), OC43 (HCoV-OC43), NL63 (HCoV-NL63), and HKU1 (HCoV-HKU1). (**e**,**f**) No cross-reactivity was observed testing Alpha and Delta VOCS. MW: Molecular weight. Bp: Base pairs. WT: Wild-type S gene (10^4^ Copies/µL). NTC: Non-template control, nuclease-free water.

**Figure 7:**
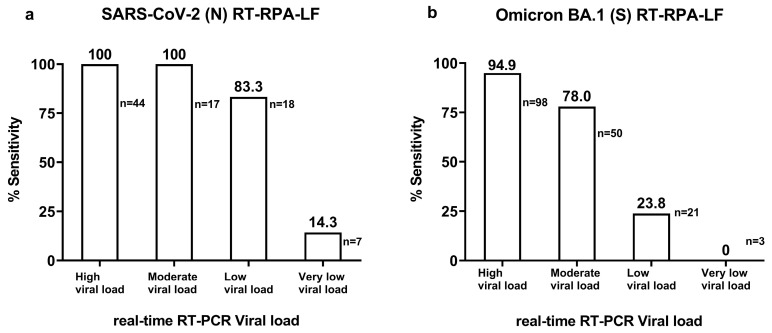
Sensitivity of SARS-CoV-2 (N) RT-RPA-LF and Omicron BA.1 (S) RT-RPA-LF tested in the clinical samples. (**a**) SARS-CoV-2 (N) RT-RPA-LF showed different sensitivities compared to real-time RT-PCR Cq values. (**b**) Omicron BA.1 (S) RT-RPA-LF showed different sensitivities compared to Cq values. Viral loads were classified in the following way. High viral load: >9015.7 copies/µL, moderate viral load: 385.5–9015.7 copies/µL, low viral load: 16.5–385.6 to copies/µL, and very low viral load: <16.5 copies/µL.

Specific RPA primers and probes were designed and selected (Appendix A) for the detection of the SARS-CoV-2 N and Omicron BA.1 spike genes (Table S1). The detection limit for the SARS-CoV-2 (N) RT-RPA-LF was 10 RNA copies/µL (Figure 5a,b), and no cross-reactivity was observed against some of the most common human respiratory viruses (Figure 6a–c). The sensitivity against the real-time RT-PCR-positive clinical samples varied according to the viral load categories as follows: 100.0% (95% CI: 98.7.1–100.0) for samples with high viral load, 100.0% (95% CI: 97.1–100.0) for those with moderate viral load, 83% (95% CI: 63.3–100.0) for those with low viral load, and 14.3% (95% CI: 0–47.4) for those with very low viral load (Figure 7a, Table S5a). We observed six false negatives under the very low viral load category, of which three resulted below the limit of detection of 10 copies/µL, while others showed 19.55, 23.25 and 53.5 copies/µL. The specificity against pre-COVID-19 samples was 100% (95% CI: 96.6–100). The detection time from reaction incubation to detection was approximately 35 min. 

Omicron BA.1 (S) RT-RPA-LF showed a detection limit of 10 RNA copies/µL (Figure 5c,d), and no cross-reactivity was observed against pre-COVID-19 coronaviruses 229E, OC43, NL63, and HKU1, as well as Alpha and Delta VOCs (Figure 6d–f). The sensitivity varied according to the real-time RT-PCR Cq categories as follows: 94.9% (95% CI: 90.3–99.8) for high viral load, 78% (95% CI: 65.5–90.5, CI = 95%) for moderate viral load, 23.8% (95% CI: 3.21–44) for low viral load, and 0% for very low viral load (Figure 7b, Table S5b). We observed three false negatives in the very low viral load category, of which two showed a viral load below the limit of detection of 10 copies/µL, and one sample was close to the limit of detection, showing 16.3 copies/µL. The specificities against the wild-type and non-BA.1 VOC samples and pre-COVID-19 samples were 96.5% (90.1–100, CI = 95%) and 95.9% (95% CI: 88.7–98.9), respectively.

The Omicron BA.1 (S) RT-RPA-LF testing yielded six false-positive results, of which three resulted from the negative group of wild-type +non-BA.1 VOC and the other three from the pre-COVID-19 group. We confirmed that there were no del211/ins214 mutations in the sequences from the samples with false-positive results in the wild-type +non-BA.1 VOC group. Three re-tested samples from the six false-positive samples yielded negative results. Therefore, we considered that the false positives occurred mainly due to technical issues during sample loading or amplicon dilution before lateral flow strips.

Among the 172 Omicron BA.1-positive samples tested, it was not possible to assess the presence or absence of del211/ins214 in five sequences; however, those samples were included in the sensitivity analysis because they were able to be classified as BA.1 lineage using the Pangolin COVID-19 Lineage Assigner. The viral load for those samples was 2.3, 19.1, 31.5, 60.4, and 1545.2 copies/µL. The frequencies of del211 and ins214 in sequences from 167 Omicron BA.1-positive samples, tested by Omicron BA.1 (S) RT-RPA-LF excluding those five samples without the sequences, were 100% (167/167) and 99.4% (164/167), respectively. The result of the sample missing the ins214 was positive by Omicron BA.1 (S) RT-RPA-LF. None of the 85 samples in the “wild-type and non-BA.1 VOC” group had del211 or ins214.

## 4. Discussion

In this study, we developed and evaluated two RPA assays for the detection of the SARS-CoV-2 partial N gene and the Omicron BA.1 variant-specific deletion–insertion mutations in the S gene. The detection limit using the RNA standard (10 copies/µL) was similar to [34] or slightly higher than that reported in other SARS-CoV-2 RT-RPA studies [35,36]. In addition, the detection limit was lower than that of other isothermal amplification methods developed for SARS-CoV-2, such as the 100 RNA copies for LAMP [37,38]. However, the RPA assays had slightly lower sensitivity than that found using real-time RT-PCR, reported by the Centers for Disease Control and Prevention, World Health Organization [39], and the Japanese National Institute for Infectious Diseases, whose detection limit is reported as one copy/µL [33].

Several previous studies have evaluated SARS-CoV-2 RT-RPA in clinical samples and reported sensitivities ranging from 65% to 100% and specificities ranging from 77% to 100% [35,36,40,41,42,43]. However, among these studies, only three included >50 SARS-CoV-2-positive clinical samples. Gosh et al. evaluated the sensitivity of different viral load categories in RT-PCR-positive samples in 76 positive clinical samples [42]. The sensitivities of RT-RPA-N for samples with Cq levels of 0–30, 31–35, and 36–40 were 97.4%, 71.4%, and 12.5%, respectively. Similarly, our study showed the high sensitivity of the SARS-CoV-2 (N) RT-RPA-LF assay in clinical samples with relatively high and moderate viral loads (both 100%). Another study reported a high sensitivity of 98.7% using RT-RPA-targeting N genes in 78 positive samples [41]. 

Although our results and those of others showed decreased sensitivity in samples with moderate and low viral loads, our two RT-RPA assays seemed to have better sensitivity than Ag-RDT [17,18] in low (N: 83%, BA.1: 24% vs. Ag: 0–14%) to moderate (N: 100%, BA.1: 78% vs. Ag: 42–86%) viral load samples (Table S7). Compared with the sensitivity of the LAMP [37], our assay also showed higher sensitivity in the limited number of low viral load samples (N: 83.3% vs. 20%), whereas the BA.1 assay showed comparable sensitivity [37] (24%) to the LAMP. The low sensitivity of LAMP on low viral samples (Cq > 30) may be due to the difficulty in reading results by color changes [44].

In other studies, the RT-RPA assay was combined with clustered regularly interspaced short palindromic repeats (CRISPR)/CRISPR-associated proteins (Cas) to improve sensitivity [45,46]. A study of CRISPR/Cas12a showed similar sensitivity to that observed in our study, and the others showed similar or slightly lower sensitivities using CRISPR/Cas12b (40 copies/µL) or CRISPR/Cas9 assays (8 copies/µL) [46], compared to our study. 

Only a few studies have applied RT-RPA with CRISPR to a large number of clinical samples. One study that evaluated 53 positive and 111 negative samples showed a high sensitivity of 96.3% and specificity of 100% [45]. Another relatively small study with 36 positive and 12 negative samples reported a sensitivity of 93.8% [24]. In this study, we did not utilize CRISPR, which usually requires a two-step system and additional incubation time. Instead, we developed an alternative, simple, and straightforward system that prioritized the time for testing. To the best of our knowledge, no study has reported the sensitivity of RT-RPA with CRISPR/Cas according to viral load levels. This may be feasible, particularly for samples with low viral loads.

Deletions and insertions have been used as molecular markers for the detection and characterization of viruses other than SARS-CoV-2 [47,48]. We selected del211/ins214 as a molecular marker since we found the del211/ins214 mutation to be highly prevalent in Omicron BA.1 during our primer design analysis. Deletions and insertions are significant sources of genetic diversification and can induce a significant impact on the properties and evolution of proteins [49]. They are also known to facilitate Alpha and Omicron viral entry mediating spike stability and immune evasion [50]. In addition, it shows an association in drug resistance for HIV-1 mediating viral reverse-transcriptase fitness [51]. Although Omicron BA.1 is not circulating anymore, we addressed the applicability of an assay to detect a SARS-CoV-2 variant containing deletions/insertions. The test design reported in this study may be useful in future applications for the detection of SARS-CoV-2 VOC using deletions/insertions as molecular markers. 

We developed a one-step system allowing the cDNA synthesis, isothermal DNA amplification, and deletion/insertion detection using an endonuclease IV activity in less than 35 min, resulting in a quicker and more straightforward method that may be an alternative to other methods already reported [24,25]. We designed a probe to bind to the remaining ends of the deletion/insertion and detect the mutation as a positive result, which is different from other PCR methods that produce negative results for deletion detection [52]. One sample with del211 but without ins214 was also positive, possibly because the probe annealed to a portion of the remaining del211 ends. Detecting specific deletions seems useful; however, these mutations may be shared with new variants in the future. Therefore, genomic monitoring of the sequence is necessary. For example, Omicron XBB.1.5, currently circulating in the United States and other countries, shares most of its spike mutations with BA.2, including a deletion mutation in del144 [53], which was previously observed in Alpha and other variants of interest.

Our study has limitations. First, clinical samples were not analyzed at the same institution using real-time RT-PCR parallel to RT-RPA-LF; therefore, the viral load was lower than the values tested at each institution. Because of RNA degradation due to time or unfreezing, some misclassification of viral load categories may have occurred. Second, the positive sample selection for Omicron BA.1 (S) RT-RPA-LF was limited to samples whose sequences were available by Sanger sequencing or NGS. Mutation-specific real-time RT-PCR, which is a highly sensitive method, allows the addition of a high number of samples with low viral loads. Third, the limited number of samples in the low and very low virus categories resulted in a wide range of 95% CI for sensitivity. Finally, RNA samples obtained from children during the pre-COVID-19 period and other VOCs were used as negative controls. Specificity was not evaluated for samples collected from populations with similar backgrounds and periods.

The RT-RPA-LF system still has some disadvantages for implementation in resource-limited settings. First, the system requires RNA extraction from RNA viruses. Therefore, additional laboratories, equipment, and time are required. Secondly, the lateral flow assay has a higher risk of contamination than the single-tube assay, which uses coloring techniques. The need to open the reaction tube after amplification to perform lateral flow detection has been highlighted as an important source of cross-contamination, owing to amplicon aerosolization [54]. 

Therefore, to utilize this sensitive detection method of specific deletion–insertion mutation in SARS-CoV-2 VOC or other RNA virus pathogens in the future, further studies may adapt the instrument-free and rapid nucleic acid extraction methods and/or single-tube systems. 

## Data Availability

The datasets generated and analyzed during the current study are available from the corresponding author upon reasonable request.

## Supplementary Materials

The following supporting information can be downloaded at: https://www.mdpi.com/article/10.3390/v15061254/s1.

## Appendix A. Primer and Probe Screening of Omicron BA.1

To evaluate the three RPA primers designed for a partial segment of the spike gene, a master mix was prepared for each primer set: Set 1, Set 2, and Set 3. The primers were evaluated using the TwistAmp Basic RPA kit. The master mixes consisted of the lyophilized pellet enzymes provided in the RPA kit, 29.5 µL of rehydration buffer, 2.4 µL of forward primer (480 nM), 2.4 µL of reverse primer (480 nM), 5 µL of template, 8.2 µL of nuclease-free water, and 2.5 µL of Magnesium Acetate (14 nM) to obtain a final volume of 50 µL. The Omicron BA.1 DNA plasmid (10^5^ copies/µL) was used as a positive control (PC), and nuclease-free water was used as a non-template control (NTC). The mixture was incubated at 37 °C for 30 min in a block heater CTU-N (Taitec Co., Ltd., Tokyo, Japan). The mixture was then homogenized using a vortex and a microcentrifuge for 3–5 min. The amplification products were resolved using 3% gel electrophoresis. Primers Sets 2 and 3 amplified a band of the expected size on the PC (Figure S1a), but Set 2 was chosen for the next step, considering a slightly more intense band; no band was observed for NTC.

Set 2 was further evaluated, and a detection limit of 10 copies/µL was found (Figure S1b). Probes 1 and 2 were assessed using primer Set 2 testing PC and NTC. Interestingly, only Probe 2 produced a band on the PC, while no band was observed on the NTC in both lateral flow strips and gel electrophoresis (Figure S1c,d). Therefore, Probe 2 was selected for subsequent evaluation.

To evaluate whether Primer Set 2/Probe 2 master mixes were prepared for Primer Set 2/Probe 2 testing PC, a negative control (NC) based on a 395 nt segment wild type for del211/ins214 (S_211del+214ins_Wildtype) (10^5^ copies/µL) (Table S2) was evaluated in triplicate with an NTC. Set 2/Probe 2 produced a band on the lateral flow strips on PC, whereas no bands were observed in any of the triplicates evaluated on the lateral flow strips on NC or NTC (Figure S1e). The amplified bands were observed by gel electrophoresis on PC and NC. A band due to the SARS-CoV02 RPA primers was expected in the NC; no band was observed in the NTC.

Finally, a combination of Primer Set 2 and Probe 2 was selected for the evaluation of clinical samples.

## Appendix B. Synthesis of RNA Standards

### Appendix B.1. SARS-CoV-2 Partial Nucleocapsid Gene

The RPA primers designed in this study for the nucleocapsid gene were modified to 22 nt, the T7 promoter sequence was inserted on the 5′ end of the forward primer, labeled as PCR_T7_N_2F and the reverse primer PCR_T7_N_2R (Table S1). The 2019-nCoVN_Positive Control (IDT; Catalog#10006625) was amplified using PCR. The master mix was composed of 0.5 µL PrimeSTAR^®^ HS DNA polymerase enzyme (2.5 U/µL) (Takara, Japan), 10 µL of 5x PrimerSTAR buffer, 4 µL of dNTP Mixture (2.5 mM), 1 µL of primer PCR_T7_N_2F (100 nM), 1 µL of reverse PCR_T7__N_2R (200 nM), 1 µL of plasmid control at 2 × 10^5^ copies/µL, and 32.5 µL of nuclease-free water. The PCR conditions were as follows: 95 °C × 10 s (98 °C × 10 s, 55 °C × 55 s, and 72 °C × 15 s) × 30 cycles. The amplification products were analyzed by 3% gel electrophoresis and purified using the QIAquick^®^ PCR Purification Kit (Qiagen, Germany). DNA concentration was determined using a Qubit^®^2.0 Fluorimeter (Invitrogen).

The amplification products were concentrated by adding 0.1 volumes of Sodium Acetate (3M) and 2.5 volumes of absolute ethanol. The mixture was incubated on ice for 20 min, followed by centrifugation at 21,300× *g* at 4 °C for 20 min. The supernatant was decanted and dried for 5 min, after which 200 µL of 75% ethanol was added. Then, the mixture was centrifuged at 21,300× *g* at 4 °C for 5 min. The supernatant was discarded and the pellet was reconstituted with 25 µL of TE buffer (10 mM Tris µHCl, pH 8.0, 1 mM EDTA) (Nippon Gene, Tokyo, Japan). DNA concentration was determined using a Qubit^®^2.0 Fluorimeter (Invitrogen, Waltham, MA, USA).

Next, 8 µL of PCR amplicon was used for the RNA transcription template using the MEGAshortscript^TM^ Kit (Ambion, Austin, TX, USA) following the manufacturer’s instructions. RNA was purified using the RNAeasy Minikit (Qiagen, Hilden, Germany) and the DNA was quantified using a Qubit^®^2.0 Fluorimeter (Invitrogen, Waltham, MA, USA).

As previously described, we calculated the number of RNA copies based on the amount of RNA, amplicon length, and Avogadro’s number. The RNA standards were aliquoted and stored at −80 °C.

### Appendix B.2. SARS-CoV-2 Partial Spike Gene

The RPA primers previously designed in this study for the partial spike gene were shortened to 22 nt, and the T7 promoter sequence was inserted in the 5′ end of the forward primer (Table S1). The DNA-positive control for Omicron BA.1 (S_211del+214 ins_deleted) was amplified by PCR using the following master mix formula: 0.5 µL of PrimeSTAR^®^ HS DNA polymerase enzyme (2.5 U/µL), 10 µL of 5x PrimerSTAR buffer, 4 µL of dNTP Mixture (2.5 mM), 1 µL of PCR_T7_214ins_SET2_F (100 nM), 1 µL of reverse PCR_T7_214ins_SET2_R (200 nM), 1 µL of plasmid control (1×10^8^ copies/µL), and 32.5 µL of nuclease-free water. The PCR conditions were 95 °C × 10 s (98 °C × 10 s, 55 °C × 55 s and 72 °C × 15 s) × 30 cycles. The amplification products were analyzed by 3% gel electrophoresis and purified using a QIAquick^®^ PCR Purification Kit (Qiagen, Hilden, Germany). The DNA concentration was determined using a Qubit^®^2.0 Fluorimeter (Invitrogen, Waltham, MA, USA).

Next, 8 µL of the PCR amplicons were employed for the RNA transcription template using the MEGAshortscriptTM Kit (Ambion, Austin, TX, USA) following the manufacturer’s recommendations. RNA was purified using the RNAeasy Minikit and quantified using a Qubit^®^2.0 Fluorimeter (Invitrogen, Waltham, MA, USA). The number of copies was calculated as described previously. The RNA standard was aliquoted and stored at −80 °C.

## Appendix C. Generation of Controls for Omicron BA.1

### Appendix C.1. Generation of Positive Control for Omicron BA.1

To generate a positive control for the Omicron BA.1 (S) RT-RPA-LF assay, we selected a 394 nt sequence ranging from 21,952 nt to 22,345 nt (OL822906.1) of Omicron BA.1 (Table S2). The sequence was cloned into a 2966 bp plasmid vector, pUCFa (FASMAC). This plasmid was utilized as a DNA-positive control and named “S_211del+214 ins_deleted”. The positive control was resuspended with 100 µL of TE buffer, and the DNA concentration was quantified using Qubit^®^2.0 Fluorimeter (Invitrogen, Waltham, MA, USA). The copy number was calculated as described elsewhere [55], and 10-fold dilutions were prepared to obtain a stock of 10^5^ copies/µL. Aliquots were prepared and stored at −80 °C until further use.

### Appendix C.2. Generation of Negative Control for Omicron BA.1

Similarly, we selected a 395 nt sequence located at 21,984–22,378 nt (OL817641.1) from SARS-CoV-2 showing a wild-type segment for the del211/ins214 mutation. The segment was cloned into the 2966 bp plasmid vector pUCFa (FASMAC). The plasmid vector was used as a DNA-negative control for Omicron BA.1 (S) RT-RPA-LF. The plasmid was resuspended in 100 µL of TE buffer. After DNA quantification, the copy number was calculated as described elsewhere [55], and 10-fold dilutions were prepared equivalent to 10^3^ copies/µL. The aliquots were stored at −80 °C until further use.
