# Supplementary material for: Rapid Detection of SARS-CoV-2 RNA Using Reverse Transcription Recombinase Polymerase Amplification (RT-RPA) with Lateral Flow for N-Protein Gene and Variant-Specific Deletion–Insertion Mutation in S-Protein Gene
Source: Viruses. 2023 May 26;15(6):1254. doi: 10.3390/v15061254 (PMC10302577; doi:10.3390/v15061254)
Supplement: Supplementary file 1 [file viruses-15-01254-s001.zip › viruses-2373212-supplementary.pdf]

Table S1. Primers and probes designed in this study

RPA primers for the detection of SARS-CoV-2 and Omicron BA.1

Primer	Nucleotide sequence (5'→3')	GC(%)	Length (nt)	Amplicon size (Bp)	Position	Genome reference
RPA_N_2F	GAACGTGGTTGACCTACACAGGTGCCATCAAAT	48.5	33	166	29203–29235	OP714083.1
RPA_N_2R-Bio	BIOTIN-GGTAAGGCTTGAGTTTCATCAGCCTTCTTCTTTT	41.2	34		29368–29335	OP714083.1
RPA_Probe_N1	FITC-ACAAAGATCCAAATTTCAAAGATCAAGTCA[THF]TTTGCTGAATAAGCA-C3	31.1	46	NA	29242–29287	OP714083.1
RPA_214ins_SET2_F	TCTTAGGGAATTTGTGTTTAAGAATATTGATGG	30.3	33	179	22072–22104	OP711808.1
RPA_214ins_SET2_R_Bio	Biotin- TATGTAAAGCAAGTAAAGTTTGAAACCTAGTG	31.3	32		22250–22219	OP711808.1
RPA_214INS_Probe_2	FITC-ACACGCCTATTATAGTGCGTGAGCCAGAA[THF] ATCTCCCTCAGGGTTT-C3	50.0	46	NA	22128–22173	OP711808.1

PCR primers adapted with the T7 promotor sequence used for the synthesis of RNA standards

PCR_T7_N_2F	*TAATACGACTCACTATAGG*GAACGTGGTTGACCTACACA	43.6	39	166	29203–29222	OP714083.1
PCR_T7__N_2R	GGTAAGGCTTGAGTTTCATCAG	45.5	22		29368–29347	OP714083.1
PCR_T7_214ins_SET2_F	*TAATACGACTCACTATAGG*GTAATTTCAAAAATCTTAG	28.1	38	185	22086–22103	ON062940.1
PCR_T7_214ins_SET2_R	TATGTAAAGCAAGTAAAGTT	25	20		22270–22251	ON062940.1

NA: Not applicable, THF: Tetrahydrofuran, FITC: Fluorescein isothiocyanate

Table S2. Consensus sequences for Omicron BA.1 and wild-type utilized for the generation of positive control (Omicron BA.1) and negative control SARS-CoV-2 (wild-type)

Name	Sequence (5′→3′)	Length (nt)	Position	Reference
S_211 del+214 ins_wildtype	AAAAGTTGGATGGAAAGTGAGTTCAGAGTTTATTCTAGTGCGAATAATTGCACTTTTGAATATGTCTCT CAGCCTTTTCTTATGGACCTTGAAGGAAAACAGGGTAATTTCAAAAATCTTAGGGAATTTGTGTTTAAG AATATTGATGGTTATTTTAAAATATATTCTAAGCACACGCCTATTAATTTAGTGCGTGATCTCCCTCAGG GTTTTTCGGCTTTAGAACCATTGGTAGATTTGCCAATAGGTATTAACATCACTAGGTTTCAAACTTTACT TGCTTTACATAGAAGTTATTTGACTCCTGGTGATTCTTCTTCAGGTTGGACAGCTGGTGCTGCAGCTTAT TATGTGGGTTATCTTCAACCTAGGACTTTTCTATTAAAATATAATGA	395	21984–22378	OL817641.1
S_211 del+214 ins_deleted	CAAAAACAACAAAAGTTGGATGGAAAGTGAGTTCAGAGTTTATTCTAGTGCGAATAATTGCACTTTTGA ATATGTCTCTCAGCCTTTTCTTATGGACCTTGAAGGAAAACAGGGTAATTTCAAAAATCTTAGGGAATTT GTGTTTAAGAATATTGATGGTTATTTTAAAATATATTCTAAGCACACGCCTATTATAGTGCGTGAGCCAG AAGATCTCCCTCAGGGTTTTTCGGCTTTAGAACCATTGGTAGATTTGCCAATAGGTATTAACATCACTAG GTTTCAAACTTTACTTGCTTTACATAGAAGTTATTTGACTCCTGGTGATTCTTCTTCAGGTTGGACAGCT GGTGCTGCAGCTTATTATGTGGGTTATCTTCAACCTAGGACTTTT	394	21952–22345	OL822906.1

Table S3. Samples used for cross-reactivity test

	Sample	Type	Cross-reactivity
**SARS-CoV-2 (N) RT-RPA-LF**	Human metapneumovirus	Clinical sample (cDNA)	No
	Influenza A virus H1N1-pandemic	Clinical sample (cDNA)	No
	Influenza C virus	Clinical sample (cDNA)	No
	Respiratory syncytial virus	Clinical sample (cDNA)	No
	Human parainfluenza virus type 1	Clinical sample (cDNA)	No
	Human parainfluenza virus type 2	Clinical sample (cDNA)	No
	Human parainfluenza virus type 3	Clinical sample (cDNA)	No
	Human parainfluenza virus type 4	Clinical sample (cDNA)	No
	Severe acute respiratory syndrome coronavirus	Bat SARS-like coronavirus isolate bat-SL-CoVZC45, genome standard (cDNA) (IDT, catalog 10006624)	No
	Middle east respiratory syndrome coronavirus	Middle East respiratory syndrome-related coronavirus isolate KNIH/002_05_2015, genome standard (cDNA) (IDT, catalog 10006623)	No
	Human coronavirus 229E	Human coronavirus 229E genome standard (ATCC-VR-740) (RNA)	No
	Human coronavirus OC43	Human coronavirus OC43 genome standard (ATCC-VR-1558) (RNA)	No
**Omicron BA.1 (S) RT-RPA-LF**	Alpha (B.1.1.7) VOC	Clinical sample (RNA)	No
	Delta (1.617.2) VOC	Clinical sample (RNA)	No
	Human coronavirus 229E	Human coronavirus 229E genome standard (ATCC-VR-740) (RNA)	No
	Human coronavirus OC43	Human coronavirus OC43 genome standard (ATCC-VR-1558) (RNA)	No
	Human coronavirus NL63	Clinical sample (RNA)	No
	Human coronavirus HKU1	Clinical sample (RNA)	No

Table S4.

Clinical samples tested using SARS-CoV-2 (N) and Omicron BA.1 (S) RT-RPA-LF

RT-RPA-LF	Kawamura children clinic[a]	Tohoku University Hospital[b]	Sendai City Institute of Public Health[c]	Virus Research Center, Sendai Medical Center[d]	Miyagi Prefectural Institute of Public Health and Environment[e]	Tohoku kosai Hospital[f]	Universidad Peruana Cayetano Heredia[g]	Total
SARS-CoV-2 (N)	104	50	28	5	3	-	-	190
Omicron BA.1 (S)	74	56	-	-	128	40	33	331

In total, 454 samples were tested in this study. Of these, 190 were tested for SARS-CoV-2 (N) RT-RPA-LF, 331 were tested for Omicron BA.1 (S) RT-RPA-LF, and 67 were tested for both. The institutions a,b,d,e, and f used the QIAamp Viral RNA Mini Kit. The institution e used the MagMAX CORE Nucleic Acid Purification Kit and Maxwell RSC Total Nucleic Acid Purification Kit. The institution g used a Nucleic Acid Extraction-Purification kit (Sansure Biotech, China). The institutions b, c, d, e, and f used the SARS-CoV-2 PCR protocol (32), (33).

Table S5

	SARS-CoV-2 PCR positive (by viral load categories)				Pre-COVID-19	Total
RT-RPA	High	Moderate	Low	Very low		
Positive	44	17	15	1	0	77
Negative	0	0	3	6	104	113
Total	44	17	18	7	104	190

(a). SARS-CoV-2 (N) RT-RPA-LF results by real-time RT-PCR using viral load categories.

	SARS-CoV-2 Omicron BA.1 (by viral load categories[a])				Non-BA.1 VOC	Pre - COVID-19	Total
RT-RPA-LF	High	Moderate	Low	Very low			
Positive	93	39	5	0	3	3	143
Negative	5	11	16	3	82	71	188
Total	98	50	21	3	85	74	331

(b). Omicron BA.1 (S) results of real-time RT-PCR using viral load categories.

VOC: Variant of concern. Viral loads were classified as high viral load : > 9,015.7 copies/µL, moderate viral load: 385.6–9,015.7 copies/µL, low viral load: 16.5–385.5 to copies/µL, and very low viral load : < 16.5 copies/µL.

Table S6.

PCR primers used for the partial amplification and sequencing of the spike protein gene of the SARS-CoV-2 using the Sanger sequencing method

Primer Name	Length (nt)	Sequence: 5'⟶3'
SARS-CoV-2_S_38F	25	GTCAGTGTGTTAATCTTACAACCAG
SARS-CoV-2_S_1191R	25	TGCATAGACATTAGTAAAGCAGAGA
SARS-CoV-2-S-omi-1017F	20	TGAAGTTTTTAACGCCACCA
SARS-CoV-2_S_2249R	24	CTGCATTCAGTTGAATCACCACAA
SARS-CoV-2-S-omi-663R	20	CGAAAAACCCTGAGGGAGAT
SARS-CoV-2-S-omi-486F	21	TGCGAATAATTGCACTTTTGA
SARS-CoV-2_S_1583R	21	TTAGGTCCACAAACAGTTGCT
SARS-CoV-2-S-omi-1363F	26	TTGTTTAGGAAGTCTAATCTCAAACC

nt = nucleotides

Table S7.

Sensitivity of some rapid assay for SARS-CoV-2 on clinical samples under different viral load values

Test	Method	Sensitivity (%) by viral load				Target
		High	Moderate	Low	Very low	
SARS-CoV-2 (N) RT-RPA-LF*	RT-RPA	44/44 (100)	17/17 (100)	15/18 (83)	1/7 (14)	SARS-CoV-2 (N)
Omicron BA.1 (S) RT-RPA-LF*	RT-RPA	93/98 (95)	39/50 (78)	5/21 (24)	0/3 (0)	Omicron BA.1 (S)
Colorimetric RT-LAMP[37]	RT-LAMP	51/51 (100)	28/30 (93)	4/20 (20)	0/16 (0)	SARS-CoV-2
BD Veritor[17]	LFAs	13/13 (100)	10/14 (71)	0/8 (0)	1/13 (8)	SARS-CoV-2
Sofia 2 SARS Ag[17]	LFAs	13/13 (100)	12/14 (86)	1/8 (13)	1/13 (8)	SARS-CoV-2
BinaxNOW[17]	LFAs	13/13 (100)	11/14 (79)	1/8 (13)	1/13 (8)	SARS-CoV-2
Standard Q COVID-19 Ag[18]	LFA	4/4 (100)	18/24 (75)	5/37 (14)	0/11 (0)	SARS-CoV-2
Espline SARS-CoV-2[18]	LFA	4/4 (100)	20/24 (83)	4/37 (11)	0/11 (0)	SARS-CoV-2
QuickNavi COVID19 Ag[18]	LFA	4/4 (100)	10/24 (42)	2/37 (5)	0/11 (0)	SARS-CoV-2

RT-LAMP: Reverse transcription loop-mediated amplification. LFA: Lateral flow antigen detection assays. N: Nucleocapsid protein gene, S: Spike protein gene. * Test developed in this study.

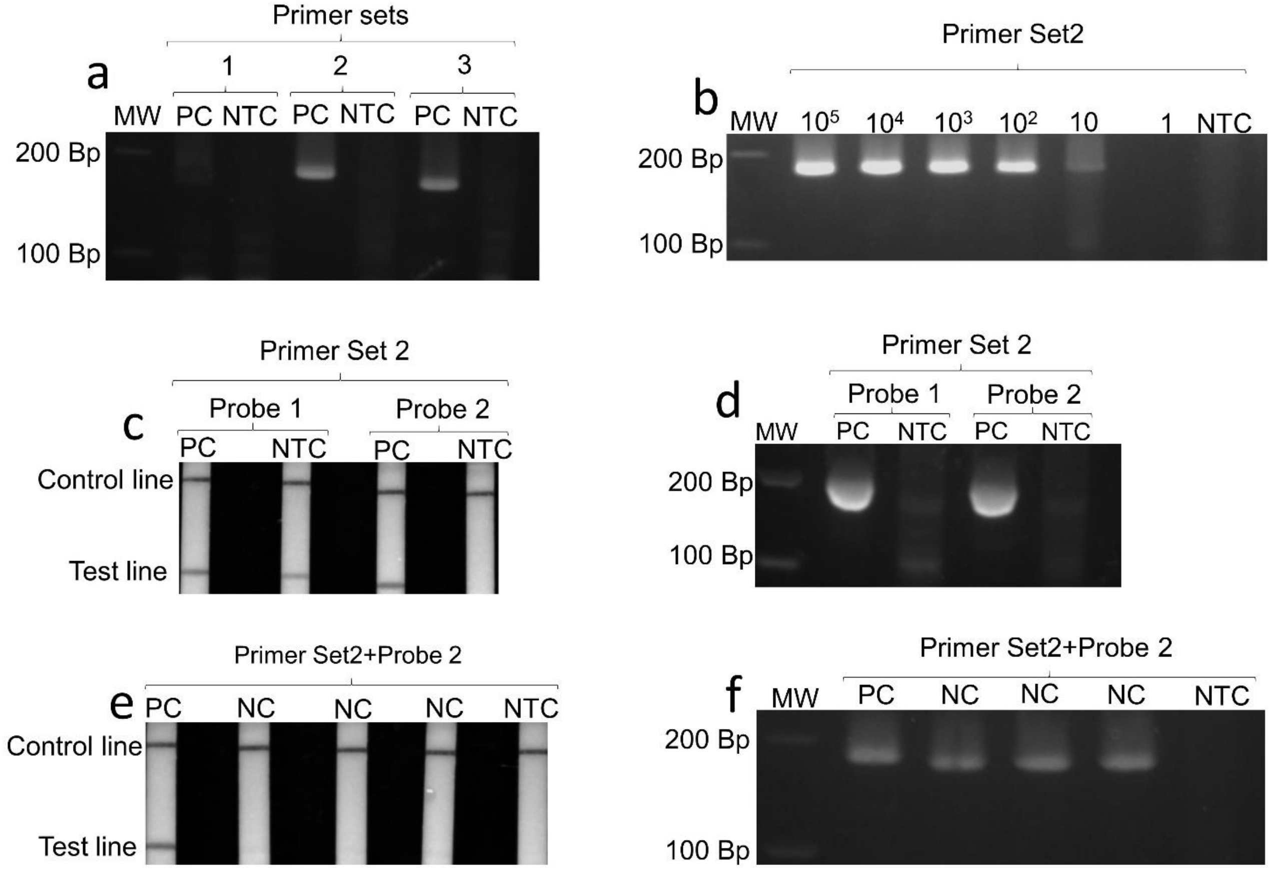

Figure S1. Primer and probe evaluation for Omicron BA.1 (S) RT-RPA-LF. **(a)** RPA primer evaluation, amplification bands are confirmed on positive control (PC) for primers Set 2 and Set 3, **(b)** gel electrophoresis shows a detection limit of 10 copies/μL (plasmid control) for primer Set 2, **(c)** the combination of primer Set 2 and Probe 2 produced a band on PC. No band was observed for the non-template control (NTC), **(d)** gel electrophoresis confirms amplification bands for both Probes 1 and 2 at the PC, but no bands were detected at the NTC. MW: molecular weight marker. Bp: base pairs. PC: positive control, del211/ins214 plasmid control 10^5 copies/μL. NC: negative control, wildtype for del211/ins214 plasmid control 10^5 copies/μL. NTC: non-template control, nuclease-free water.
